# D-arginine Enhances the Effect of Alpha-Amylase on Disassembling *Actinomyces viscosus* Biofilm

**DOI:** 10.3389/fbioe.2022.864012

**Published:** 2022-03-03

**Authors:** Baosheng Li, Qing Cai, Zixuan Wang, Shuwei Qiao, Yanzhen Ou, Rui Ma, Chuanfu Luo, Weiyan Meng

**Affiliations:** ^1^ Department of Dental Implantology, Hospital of Stomatology, Jilin University, Changchun, China; ^2^ Jilin Provincial Key Laboratory of Oral Biomedical Engineering, Changchun, China; ^3^ Xinjiang Laboratory of Phase Transitions and Microstructures in Condensed Matters, College of Physical Science and Technology, Yili Normal University, Yining, China; ^4^ School of Applied Chemistry and Engineering, University of Science and Technology of China, Hefei, China

**Keywords:** D-arginine, alpha-amylase, *Actinomyces viscosus*, biofilm, exopolysaccharides, molecular dynamics, molecular docking

## Abstract

Peri-implantitis is the leading cause of dental implant failure, initially raised by biofilm accumulation on the implant surface. During the development of biofilm, *Actinomyces viscosus* (*A. viscosus*) plays a pivotal role in initial attachment as well as the bacterial coaggregation of multispecies pathogens. Hence, eliminating the *A. viscosus*-associated biofilm is fundamental for the regeneration of the lost bone around implants. Whereas clinical evidence indicated that antimicrobials and debridement did not show significant effects on the decontamination of biofilm on the implant surface. In this study, alpha-amylase was investigated for its effects on disassembling *A. viscosus* biofilm. Then, in order to substantially disperse biofilm under biosafety concentration, D-arginine was employed to appraise its enhancing effects on alpha-amylase. In addition, molecular dynamics simulations and molecular docking were conducted to elucidate the mechanism of D-arginine enhancing alpha-amylase. 0.1–0.5% alpha-amylase showed significant effects on disassembling *A. viscosus* biofilm, with definite cytotoxicity toward MC3T3-E1 cells meanwhile. Intriguingly, 8 mM D-arginine drastically enhanced the eradication of *A. viscosus* biofilm biomass by 0.01% alpha-amylase with biosafety in 30 min. The exopolysaccharides of biofilm were also thoroughly hydrolyzed by 0.01% alpha-amylase with 8 mM D-arginine. The biofilm thickness and integrity were disrupted, and the exopolysaccharides among the extracellular matrix were elusive. Molecular dynamics simulations showed that with the hydrogen bonding of D-arginine to the catalytic triad and calcium-binding regions of alpha-amylase, the atom fluctuation of the structure was attenuated. The distances between catalytic triad were shortened, and the calcium-binding regions became more stable. Molecular docking scores revealed that D-arginine facilitated the maltotetraose binding process of alpha-amylase. In conclusion, these results demonstrate that D-arginine enhances the disassembly effects of alpha-amylase on *A. viscosus* biofilm through potentiating the catalytic triad and stabilizing the calcium-binding regions, thus providing a novel strategy for the decontamination of biofilm contaminated implant surface.

## Introduction

Dental implants have become the primary protocol for the treatment of dentition defects and edentulous patients nowadays. Despite its high success rates, complications could reduce the long-term cumulative implant survival (Fu and Wang, 2020). Peri-implantitis (PiM), as the primary cause leading to implant failure, could arise about 22% in 10 years after implantation (Derks and Tomasi, 2015). During PiM pathogenesis, the inflammation at the implant-bone interface is evoked to successively cause bone resorption, gingival tissue retraction, and implant surface exposure. To date, plaque biofilm is a putative and pivotal factor that induces the initial inflammation of PiM (Berglundh et al., 2018). Hence, eliminating biofilm is essential for the prevention and treatment of PiM.

In clinical practice, methods for decontaminating the implant surface could be divided into physical and chemical approaches. Physical approaches, such as mechanical scaling, sandblasting, magnetic nanoparticles, and laser therapy, could damage the biological surface, cause material residue, or be difficult to eliminate the massive biofilm matrix enmeshed in the micropores due to the rough character of the implant surface (Elbourne et al., 2020; Lasserre et al., 2020; Vyas et al., 2020). Correspondingly, chemical approaches, including antimicrobial agents, antimicrobial peptides, and photodynamic therapy (PDT), et al., mainly focus on eradicating bacteria or disturbing biofilm’s integrity (Chen and Lee, 2018; Liu et al., 2019; Pinto et al., 2020). Whereas the biofilm extracellular matrix, mainly composed of exopolysaccharides (EPS), lipids, proteins, and extracellular DNA (eDNA), represents around 90% of the total biofilm biomass (Fulaz et al., 2019). Consequently, even if the bacteria were thoroughly damaged, the massive biofilm matrix remains attached to the implant surface. Nevertheless, the extracellular matrix protects bacteria from agents penetration and shear stress, making a low bioavailability of agents’ germicidal effects (Flemming and Wingender, 2010). Previous *in vivo* studies have demonstrated that there was fibrous connective tissue between regenerated bone and exposed implant surface with PiM after grafting autogenous bone grafts (You et al., 2007), indicating that biofilm-associated contaminants obstructed the re-osseointegration of the implant surface. Till now, the methods for treating PiM are unpredictable yet. Thus, strategies for eradicating the whole biofilm should be prospective.

The biofilm extracellular matrix constitutes the main component of biofilm, while EPS are one of the most constituents of the extracellular matrix (Pinto et al., 2020). Hence, EPS is indispensable to biofilm formation and constitutes the protective barrier of encapsulated bacteria (Flemming and Wingender, 2010). Besides, EPS occupies the main adhesion force binding to the biological surface as well as bacteria and proteins through Van der Waals force, electrostatic attraction, hydrogen bonds (Arciola et al., 2018), thus facilitating the cohesion of the biofilm structure. In addition, EPS is responsible for water retention within the biofilm, and provides nutrient sources and ions for the inner bacteria (Pinto et al., 2020). Therefore, EPS could be a potential target for eradicating the biofilm on implant surface.

Similar to plaque biofilm on teeth, the biofilm formation on implant surface possesses typical stages, namely initial, early, secondary, and late colonizations (Vilarrasa et al., 2018; Bermejo et al., 2019). *Streptococcus oralis* and *Actinomyces viscosus* (*A. viscosus*) are the representative strains in the initial stage, then *Veillonella parvula* in the early stage, *Fusobacterium nucleatum* in the secondary stage, and *Porphyromonas gingivalis* in the late stage. In fact, *Fusobacterium nucleatum* and *Porphyromonas gingivalis* could only produce capsular polysaccharides but not EPS (Davey and Duncan, 2006), while *Veillonella* species utilize EPS secreted by *Streptococci* to adhere to biofilm (Liu et al., 2020). In addition, in the study of multispecies biofilms growing on the implant surface, *Actinomyces* showed about two-fold amount of biomass than *Streptococcus* (Bermejo et al., 2019). Moreover, *Actinomyces* plays an essential role as physical bridges to mediate coaggregation and coadhesion between coaggregating partners, which occurs 5- to 10-fold more often than coadhesion between noncoaggregating cells (Kolenbrander, 2000). Thus, disintegrating the *Actinomyces* EPS and the *Actinomyces*-associated biofilm is critical for the decontamination of the implant surface.

EPS could be composed of glucose, mannose, galactose, N-acetyl-glucosamine, and other monosaccharides (Rabin et al., 2015), which could link as α-1,4 bond, β-1,4 bond, or β-1,3 bond. Glycoside hydrolases are enzymes that hydrolyze the glycosidic linkages between carbohydrates within polysaccharides or oligosaccharides (Naumoff, 2011). *α*-amylase (Amy) and cellulase effectively disrupted *Staphylococcus aureus* and *Pseudomonas aeruginosa* coculture biofilms by breaking down complex polysaccharides. However, whether glycoside hydrolases could disrupt *A. viscosus* biofilm remains unknown.

Our previous studies demonstrated that D-arginine (R) could disperse *Porphyromonas gingivalis* mature biofilm in 72 h (Li et al., 2020; Zhang et al., 2021). Whereas the treatment time takes too long for feasible clinical practice. The capability of R to disrupt *A. viscosus* biofilm and higher treatment efficiency should be further tested.

Therefore, the aim of this study was to investigate the effects of Amy on disassembling *A. viscosus* biofilm and the enhancing effects of R on Amy. Molecular dynamics (MD) simulations and molecular docking were furtherly exploited to explore the intrinsic mechanisms.

## Materials and Methods

### Disassembly Effects of Multiple Glycoside Hydrolases on *A. viscosus* Biofilm

*A. viscosus* ATCC 27044 was used in this study. Briefly, *A. viscosus* was subcultured on sterilized brain heart infusion (BHI) (HopeBio, Qingdao, China) supplemented with yeast extract (LP0021, Oxoid), menadione (0.5 μg/ml), hemin (5 μg/ml), and sucrose (0.5 μg/ml), then incubated aerobically at 37°C (80% N_2_, 10% H_2_, and 10% CO_2_). Subsequently, single *A. viscosus* colonies were inoculated into BHI bacterium liquid medium, then adjusted to 10^8^ colony-forming unit counts/ml (CFU/ml) for inoculation to 24-well plates, then incubated for 48 h at 37°C under anaerobic conditions to form mature biofilm. Subsequently, the bacterial supernatant was removed and the culture plate was rinsed with phosphate-buffered saline (PBS) for 3 times. Afterward, 500 μl of the following different glycoside hydrolases were added to each well, respectively: 0.5% (w/v) Amy (Yuanye, Shanghai, China), 0.5% (w/v) cellulase (Yuanye, Shanghai, China), 0.5% (w/v) dextranase (Yuanye, Shanghai, China), 1% (v/v) *α*-galactosidase (Yuanye, Shanghai, China), 1% (v/v) *β*-galactosidase (Yuanye, Shanghai, China), and 1% (v/v) Dispersin B (Sigma-Aldrich, St Louis, MO, United States). All glycoside hydrolases were prepared by dissolving lyophilized powder or stock solution in double-distilled water (ddH_2_O). 500 μl PBS was used as the control group. After 30 min of treatment at 37°C, crystal violet (CV) assay was carried out as referred to the previous study (Zhang et al., 2021). Finally, the biofilm biomass was evaluated at 595 nm using a microplate reader (Synergy HT, BioTek, Winooski, VT, United States).

### Disassembly Effects of Gradient Concentrations of Amy on *A. viscosus* Biofilm

*A. viscosus* mature biofilm was cultured same to 2.1. Then, gradient concentrations of Amy (0.01, 0.05, 0.1, 0.25, and 0.5%) were employed to investigate their effects on disassembling *A. viscosus* mature biofilm. In order to stabilize Amy, 60 ppm of CaCl_2_ was added to each group (The following experiments were the same). PBS was used as the control group.

### Cytotoxicity Assay

Murine pre-osteoblast MC3T3-E1 cells were resuscitated and then cultured in DMEM solution, placed in an incubator of 5% CO_2_ at 37°C. Subsequently, the logarithmic growth phase cells were inoculated in 96-well plates with 0.01% Amy, 0.05% Amy, 0.1% Amy, 0.25% Amy, 0.5% Amy, 2 mM R, 4 mM R, 8 mM R, 16 mM R, and 0.01% Amy + 8 mM R, respectively. Isochoric DMEM was used as the control group. After 1 and 3 days of culture at 37°C, 10 μl CCK-8 (Beyotime, Shanghai, China) was added to each well and sequentially cultured for 2 h at 37°C. The absorbance value was measured at 450 nm.

### Effects of R on Interfering Amy Disassembling *A. viscosus* Biofilm

In order to substantially disperse *A. viscosus* biofilm under biosafety concentration, R was employed. Briefly, R was mixed with Amy to form the following concentration ratios: 0.01% Amy + 1/2/4/8 mM R. In addition, mono 1/2/4/8/16 mM R and 0.01% Amy were also determined. The PH of the groups containing R was adjusted to 7.0 by HCl. PBS was used as the control group. 500 μl of the groups mentioned above was added to each well to disrupt *A. viscosus* mature biofilm. Subsequently, in order to verify the component orchestrating the catalytic role, 0.01% Amy or 8 mM R was respectively heat-inactivated by heating the solutions at 95°C for 5 min. PBS was used as the control group. Furtherly, in order to determine an optimal treatment time, 0.01% Amy + 8 mM R was tested for 10, 20, 30, and 60 min.

### Effects of R on Enhancing Amy Hydrolyzing Exopolysaccharides

After treatment with 0.01% Amy or 8 mM R or 0.01% Amy + 8 mM R for 30 min, each well was rinsed with 1 ml PBS, the suspension containing dispersed biofilm and agents was collected to the EP tube, centrifuged at ×12,000 g (Centrifuge 5810 R, Eppendorf, Framingham, MA, United States) for 5 min (4°C) to collect the hydrolyzed exopolysaccharides (HEPS) in the supernatant. Thereafter, the residual biofilm was scraped carefully with a cell scraper (Nunc, Thermo Fisher Scientific, Waltham, MA, United States), rinsed with 1 ml PBS, and then centrifuged at ×12,000 g for 5 min (4°C). After removing the supernatant, 200 μl NaOH was added to dissolve the sediment, then centrifuged again. The supernatant was collected as the unhydrolyzed exopolysaccharides (UEPS) of biofilm. Furtherly, in order to compare the enhancing effects between R and Ca^2+^ on Amy, a soluble starch (Sigma-Aldrich, St. Louis, MI, United States) was employed. Briefly, the starch was dissolved in boiling ddH_2_O by 2% (w/v). The experimental groups were: 0.01% Amy, 0.01% Amy + 60 ppm CaCl_2_, 0.01% Amy + 8 mM R, 0.01% Amy + 60 ppm CaCl_2_ + 8 mM R. Isochoric ddH_2_O was used as the control group. 10 μl of each group was added to 1 ml starch solution. After treatment for 3 min at 37°C, the reaction was terminated by heating the solutions at 95°C for 5 min. All above specimens were determined by the DNS method at 540 nm with glucose as standard (Wu et al., 2018).

### Confocal Laser Scanning Microscopy Assay

The effects of 0.01% Amy and 0.01% Amy + 8 mM R on *A. viscosus* biofilm as well as EPS were assessed by Confocal Laser Scanning Microscopy (CLSM). SYTO9 (Invitrogen, Waltham, MA, United States), SYPRO Ruby (Invitrogen, Waltham, MA, United States), and Calcofluor (Sigma-Aldrich, St. Louis, MI, United States) were employed for bacteria labeling, protein labeling, and EPS labeling, respectively. In general, biofilm was cultured for 48 h on confocal dishes (WHB, Shanghai, China). Followed by the treatment of the above agents for 30 min and subsequent rinsing with PBS. 200 μl SYTO9, 200 μl SYPRO Ruby, and 15 μl Calcofluor was added to each well simultaneously, then incubated for 30 min at 20°C in a dark area. Afterward, the specimens were detected by a confocal laser scanning microscope (FV3000, Olympus, Japan), with a green channel (480/500 nm) for SYTO9, a red channel (450/610 nm) for SYPRO Ruby, and a blue channel (365/450 nm) for Calcofluor. Images were captured by Imaris software (Zeiss, Germany). The thicknesses and biomass of the biofilm and EPS and the surface to biovolume ratio were calculated using ImageJ COMSTAT2 software (Heydorn et al., 2000).

### Scanning Electron Microscopy Analysis

The biofilms in 24-well plates were treated with 0.01% Amy + 0/1/2/4/8 mM R for 30 min. After rinsing with PBS, the specimens were fixed overnight in 2.5% (v/v) glutaraldehyde at 4°C and dehydrated by gradient ethanol solutions (30/50/70/80/90/95/100%). The biofilm without any treatment was used as the control group. Finally, the biofilms were observed under Scanning Electron Microscopy (SEM) (Merlin, Zeiss, Germany) after oven drying and gold sputter coating.

### Transmission Electron Microscopy (TEM) Analysis

The biofilms in 24-well plates were treated with 0.01% Amy + 8 mM R for 30 min. After rinsing with 1 ml PBS, the suspension was collected as the dispersed biofilm. While the residual biofilm on the plate was scraped and then rinsed with 1 ml PBS, the suspension was collected as the undispersed biofilm. The biofilm without any treatment was used as the control group. The specimens were centrifuged at 4,000 rpm (Centrifuge 5810R, Eppendorf, Framingham, MA, United States) for 10 min (4°C). The following preparation process of samples was performed according to previous studies (Lukic et al., 2020; Zhang et al., 2021). Finally, the ultrathin sections were observed under a transmission electron microscope (JEM 1400 PLUS, JEOL, Akishima, Japan, United States).

### Molecular Dynamics Simulations

In order to elucidate the mechanisms of R enhancing the effects of Amy hydrolyzing polysaccharides, Molecular Dynamics (MD) simulations and molecular docking were furtherly conducted. In general, the investigated Amy crystal structure from *Bacillus Subtilis* was retrieved from the protein data bank (PDB) under the code 1UA7 (Kagawa et al., 2003). MD simulations were executed using the GROMACS package version 2019.5 (Abraham et al., 2015) under constant temperature/pressure and periodic boundary conditions. Amber99sb and SPC were selected as the all-atomic force field and the water model, respectively. During the MD simulation, all covalent bonds involving hydrogen atoms were constrained by LINCS algorithm, and the integration time step was 2 fs. The electrostatic interaction was calculated using the particle-mesh Ewald (PME) method with the cutoff value set to 1.0 nm. The cutoff value of non-bond interaction was set to 10 Å, updated every 10 steps. The V-rescale temperature coupling method was used to control the simulation temperature to 300 K, while the Parrinello-Rahman method was employed to control the pressure to 1 bar. Firstly, an energy minimization step was conducted using the Steepest Descent algorithm. Then, canonical ensemble (or substance-volume-temperature, NVT) balance and isothermal-isobaric ensemble (or substance-pressure-temperature, NPT) balance simulation were carried out for 100 ps at 300 K. Afterward, 10 molecules of R were added to the system, followed by adding Na^+^ and Cl^−^ ions proportionally to neutralize the system. Finally, MD was carried out with conformations saved per 10 ps, totally simulation for 500 ns. The results were visualized and analyzed using PyMOL version 2.5.2.

### Molecular Docking

The three-dimensional structure of maltotetraose was retrieved and then dissociated from PDB under the code 1QPK(Hasegawa et al., 1999). Then, maltotetraose was selected as the ligand, while Amy and Amy + 10 R were selected as receptors, respectively. The structures of ligand and protein were imported into AutoDockTools-1.5.7 software for adding hydrogens and calculating the total charge. After defining a box center at the central point of the catalytic triad, molecular docking was carried out *via* AutoDock. Afterward, the conformations were visualized using PyMOL, and the docking scores were finally analyzed.

### Statistical Analysis

Data were expressed as mean ± standard deviation. One-way analysis of variance (ANOVA) with appropriate post-tests was employed using SPSS 24.0 software (SPSS Inc., Chicago, IL, United States). Values of *p* < 0.05 were considered statistically significant.

## Results

### Effects of Glycoside Hydrolases on Disassembling *A. viscosus* Biofilm

**FIGURE 1:**
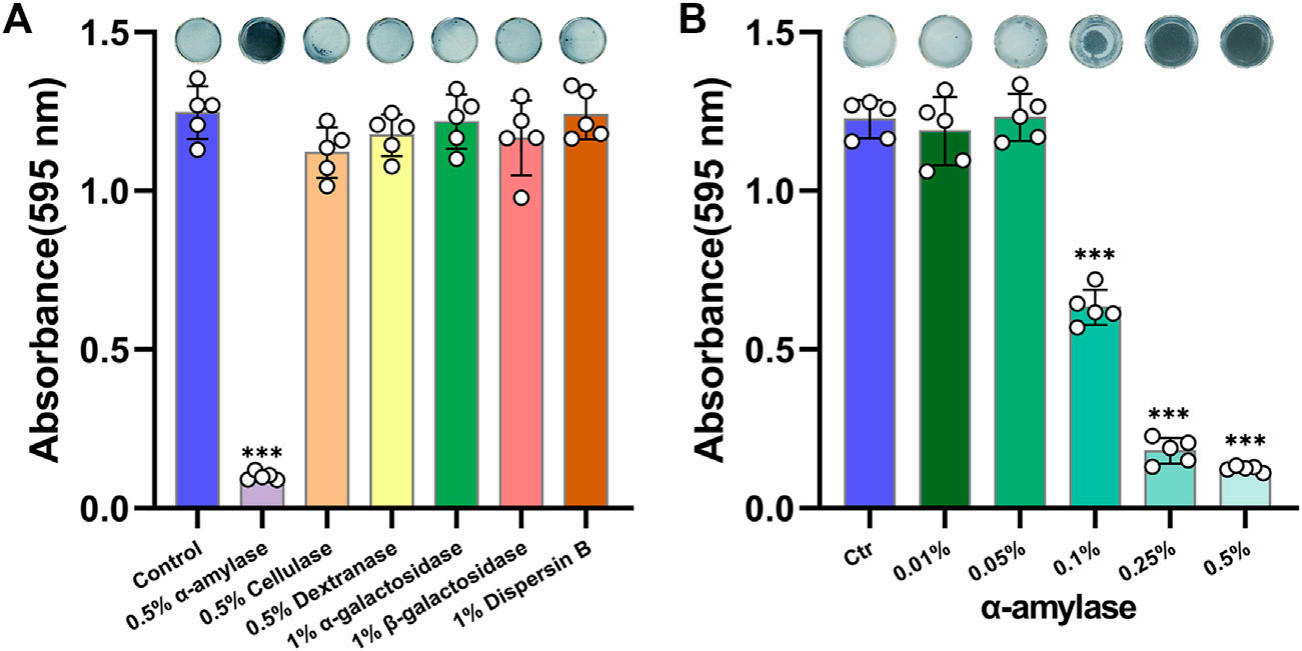
Effects of glycoside hydrolases on disassembling *A. viscosus* biofilm. **(A)** The efficiency of six different glycoside hydrolases on dispersing *A. viscosus* biofilm. **(B)** Disassembly effects of gradient concentrations of Amy on *A. viscosus* biofilm. Data represented as means ± S.D. (*n* = 5). ***p < 0.001, vs. control group. Ctr, control.

The availabilities of multiple glycoside hydrolases were investigated firstly. As shown in Figure 1A, 0.5% Amy showed a sharp effect on disassembling *A. viscosus* biofilm, whereas, other glycoside hydrolases had no significant effects. Subsequently, gradient concentrations of Amy were assessed. 0.1, 0.25, and 0.5% Amy showed statistical differences compared with the control group, while 0.01 and 0.05% Amy could not disperse the *A. viscosus* mature biofilm effectively (Figure 1B).

### Cytocompatibility

**FIGURE 2:**
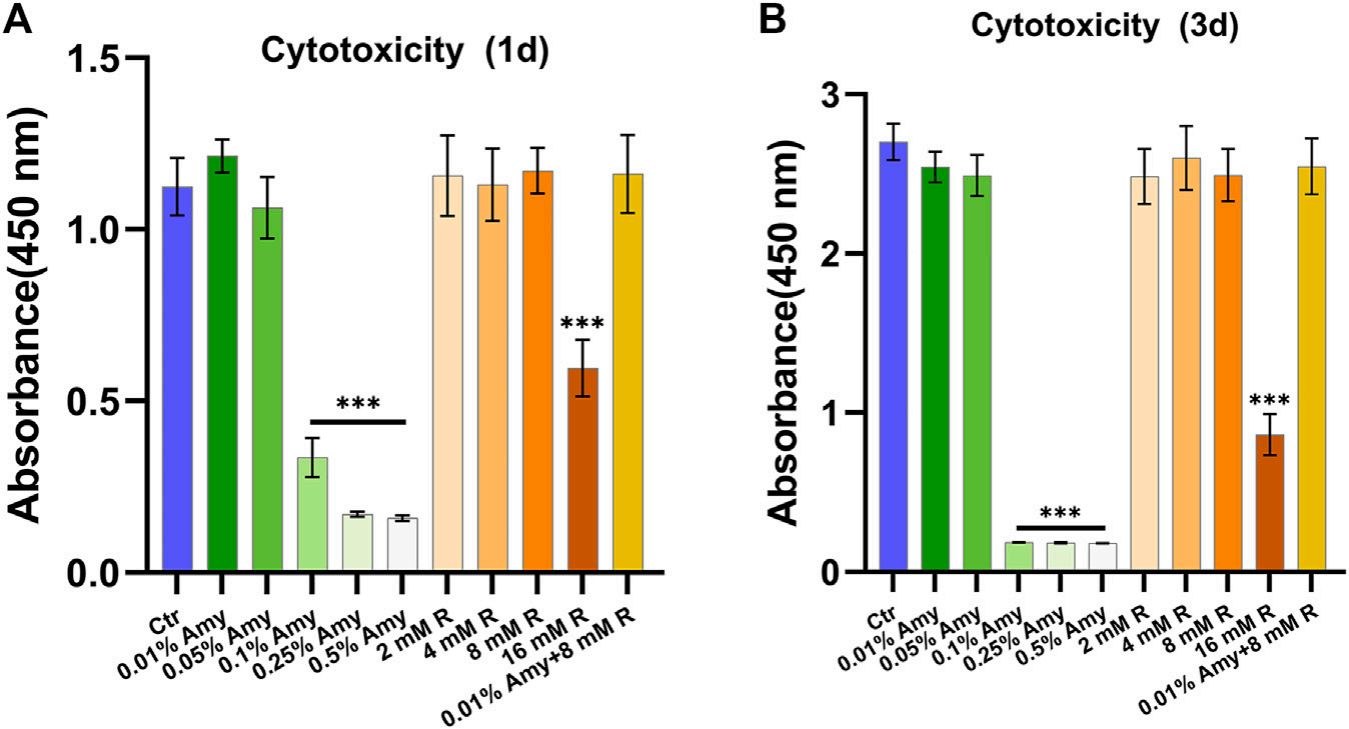
Cytotoxicity of gradient concentrations of Amy, R, and the compounds to murine pre-osteoblast MC3T3-E1 cells. **(A)** Cell viability at day 1. **(B)** Cell viability at day 3. Data represented as means ± S.D. (*n* = 5). ***p < 0.001, vs. control group. Ctr, control. Amy, *α*-amylase. R, D-arginine.

As shown in Figure 2, 0.1, 0.25, 0.5% Amy, and 16 mM R had evident cytotoxic effects towards MC3T3-E1 cells both at 1d and 3d. Correspondingly, 0.01%, 0.05% Amy, 1/2/4/8 mM R, and 0.01% Amy + 8 mM R had good biocompatibility with MC3T3-E1 cells. Hence, the groups without cytotoxicity were investigated in the following experiments.

### R Enhanced the Effects of Amy on Disassembling *A. viscosus* Biofilm

**FIGURE 3:**
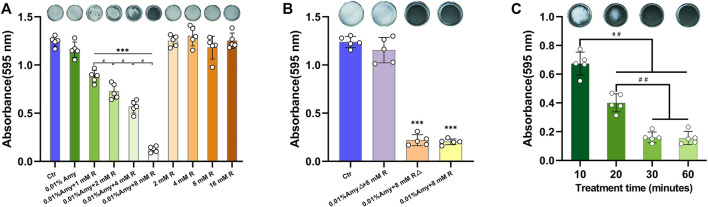
Effects of R on interfering Amy disassembling *A. viscosus* biofilm. **(A)** Effects of different compounds on disassembling *A. viscosus* biofilm. **(B)** Catalytic component determination by heat-inactivation. **(C)** Disassembly efficiency of different treatment time of 0.01% Amy + 8 mM R on *A. viscosus* biofilm. Data represented as means ± S.D. (*n* = 5). ***p < 0.001, vs. control group. #, *p* < 0.05. ##, *p* < 0.01. Δ, heat-inactivation at 95°C for 5 min. Ctr, control. Amy, *α*-amylase. R, D-arginine.

Since 0.01% is the saturation concentration of Amy, the higher concentrations were excluded in the following experiments. Intriguingly, 0.01% Amy obtained the feasibility of disassembling *A. viscosus* biofilm with the addition of 1/2/4/8 mM R, being a concentration-dependent behavior (Figure 3A). Particularly, 0.01% Amy + 8 mM R showed the most striking result. Meanwhile, 1/2/4/8 mM R failed to disperse *A. viscosus* biofilm solely, even at the cytotoxic concentration of 16 mM. Supplementary Video S1 recorded the dispersing efficiency of the control, 0.01% Amy, and 0.01% Amy + 8 mM R groups after treatment for 30 min. Supplementary Figure S1 showed that 1/2/4/8 mM R also presented a similar concentration-dependent behavior on enhancing 0.005% Amy, whereas there remained a considerable amount of biofilm in the 0.005% Amy + 8 mM R group. Figure 3B shows that the heat-inactivated 0.01% Amy lost its activity in spite of the addition of 8 mM R. While the same heat-inactivation method did not impede the enhancing effect of 8 mM R on Amy, which showed equivalent behavior compared with none-heated 0.01% Amy + 8 mM R. Moreover, 30 and 60 min of treatment showed better results than 10 and 20 min, respectively. Whereas 60 min did not show a significant difference compared with 30 min (Figure 3C).

### R Enhanced Amy Hydrolyzing Exopolysaccharides

**FIGURE 4:**
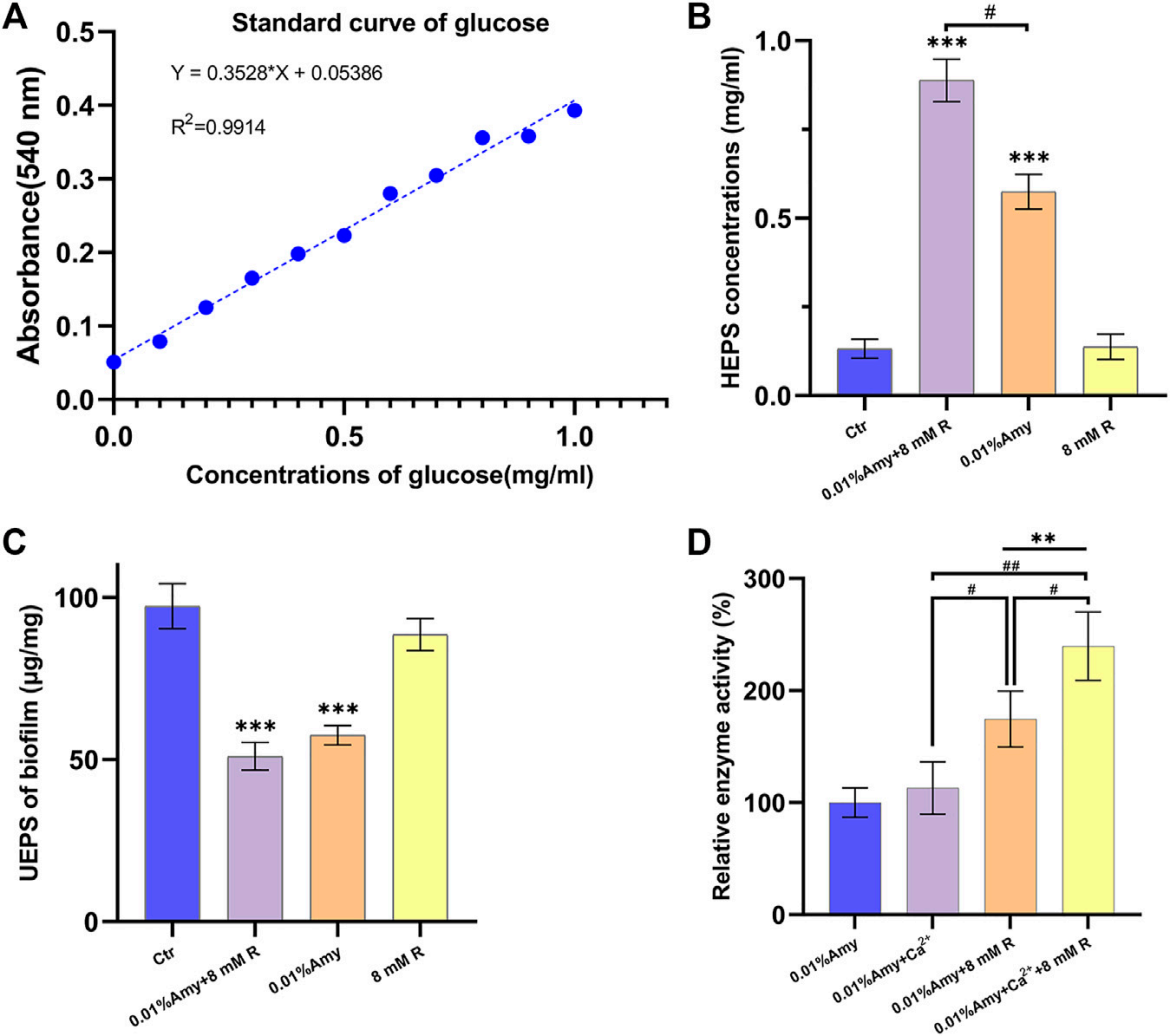
Effects of R on enhancing Amy hydrolyzing *A. viscosus* biofilm EPS and starch. **(A)** Standard curve of glucose. **(B)** The concentrations of HEPS in the dispersed biofilm after treatment for 30 min. **(C)** The contents of UEPS remained on the plate after treatment for 30 min. **(D)** Comparison of 60 ppm Ca^2+^ and 8 mM R on enhancing 0.01% Amy hydrolyzing starch. Data represented as means ± S.D. (*n* = 5). **p < 0.01, *vs*. control group. ***p < 0.001, vs. control group. #, *p* < 0.05. ##, *p* < 0.01. Ctr, control. Amy, *α*-amylase. R, D-arginine. HEPS, hydrolyzed exopolysaccharides. UEPS, unhydrolyzed exopolysaccharides.

The standard curve of glucose was generated to define the contents of EPS (Figure 4A). As shown in Figure 4B, the HEPS of 0.01% Amy + 8 mM R and 0.01% Amy were significantly higher than the control group as well as the 8 mM R group. In addition, 0.01% Amy + 8 mM R showed a better effect compared with 0.01% Amy. The value of UEPS in Figure 4C was calculated from the EPS weight within the biofilm divided by the biofilm weight. The results indicated that the remained EPS on the plate of 0.01% Amy + 8 mM R group and 0.01% Amy group was less than the control group and the 8 mM R group, but there was not a statistics difference between the 0.01% Amy + 8 mM R and the 0.01% Amy group.

In order to compare the stabilizing and promoting effects of R and Ca^2+^on Amy, R or Ca^2+^ was added respectively or simultaneously. As shown in Figure 4D, there was no significant increase of Amy hydrolyzing starch with the addition of Ca^2+^. While 8 mM R showed a definite increase compared with 0.01% Amy solely or 0.01% Amy + Ca^2+^. Furthermore, 0.01% Amy + Ca^2+^ + 8 mM R showed the most notable effects compared with the above groups.

### Thickness and Biomass of *A. viscosus* Biofilm and EPS

**FIGURE 5:**
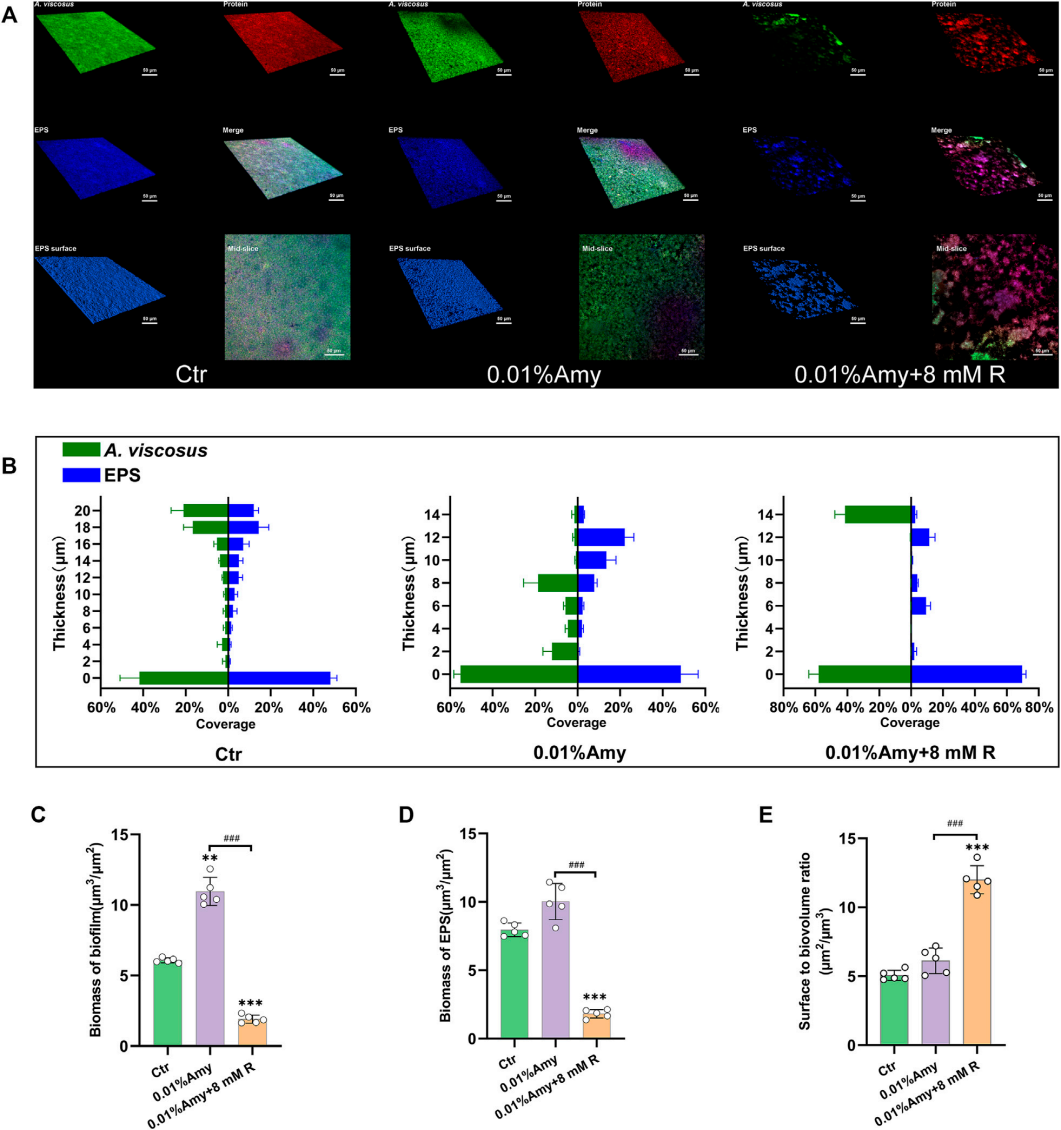
Quantification of biofilm and EPS of *A. viscosus via* CLSM analysis. **(A)** Three-dimensional visualization of *A. viscosus* biofilm cultured on confocal dishes. Live bacteria, protein, and EPS were green-labeled, red-labeled, and blue-labeled, respectively. The fourth, fifth, and sixth image of each group represent the merged labeled image, the EPS surface, and the mid-slice along the thickness-axis of biofilm, respectively. **(B)** Quantification of the thickness of biofilm and EPS of *A. viscosus*. **(C)** The biomass of biofilm of each group. **(D)** The biomass of EPS of each group. **(E)** The surface to biovolume ratio of each group. Data represented as means ± S.D. (*n* = 5). **p < 0.01, vs. control group. ***p < 0.001, vs. control group. ###, *p* < 0.001. Scale bars: 50 μm. Ctr, control. Amy, *α*-amylase. R, D-arginine.

The CLSM determined the three-dimensional structure of *A. viscosus* biofilm and EPS distribution. As shown in Figure 5A, the bacteria amount, protein content, and EPS biomass were evenly massive in the control group. While the compactness of biofilm in the 0.01% Amy group seemed attenuated, discrete voids could be observed in the mid-slice image. Interestingly, the bacteria, protein, and EPS were drastically sparse in the 0.01% Amy + 8 mM R group. The EPS surface was discontinuous with extensive voids.

Figure 5B indicates that the volume of biofilm and EPS in the control group was consistently thick. With the treatment of 0.01% Amy, the biofilm became dispersed, presented as the reduction of thickness. 0.01% Amy + 8 mM R thoroughly attenuated the thickness and distribution of biofilm as well as EPS. Only 14 μm of biofilm could be measured. Whereas the thickness of EPS was not strictly consistent with biofilm.

Figures 5C,D show that the biomass of biofilm and EPS in the 0.01% Amy + 8 mM R group were significantly less than the control group and the 0.01% Amy group. Unexpectedly, the biofilm biomass in the 0.01% Amy group was more than the control group.

The surface to biovolume ratio of the 0.01% Amy + 8 mM R group was much higher than the control group and the 0.01% Amy group (Figure 5E).

### *A. viscosus* Biofilm Morphology

**FIGURE 6:**
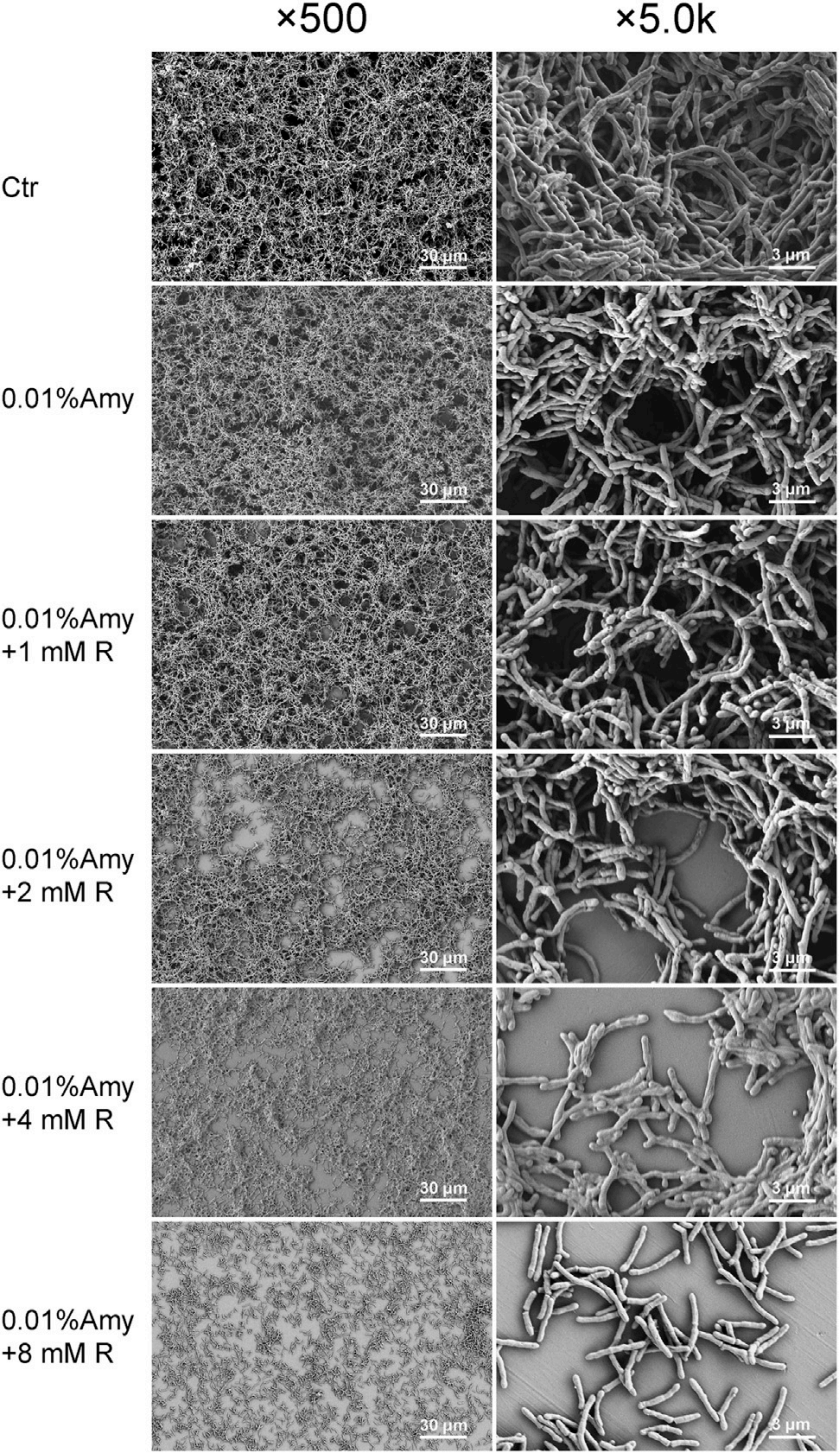
Effects of Amy and R on *A. viscosus* biofilm morphology under SEM. The left column represents ×500 magnification, the right column represents ×5.0 k magnification. Scale bars: 30 μm in the left column, 3 μm in the right column. Ctr, control. Amy, *α*-amylase. R, D-arginine.

As shown in Figure 6, the biofilm in the control group was compact and multi-layered. While 0.01% Amy seemed to loosen the biofilm. With the addition of R by gradient increase of concentrations, the integrity of biofilm was gradually disrupted and the thickness was also diminished. Particularly, the cells in 0.01% Amy + 8 mM R group tended to be sparse, and the extracellular matrix was elusive. The results were consistent with the analysis of CLSM (Figure 5).

### Structure of *A. viscosus* Biofilm Extracellular Matrix

**FIGURE 7:**
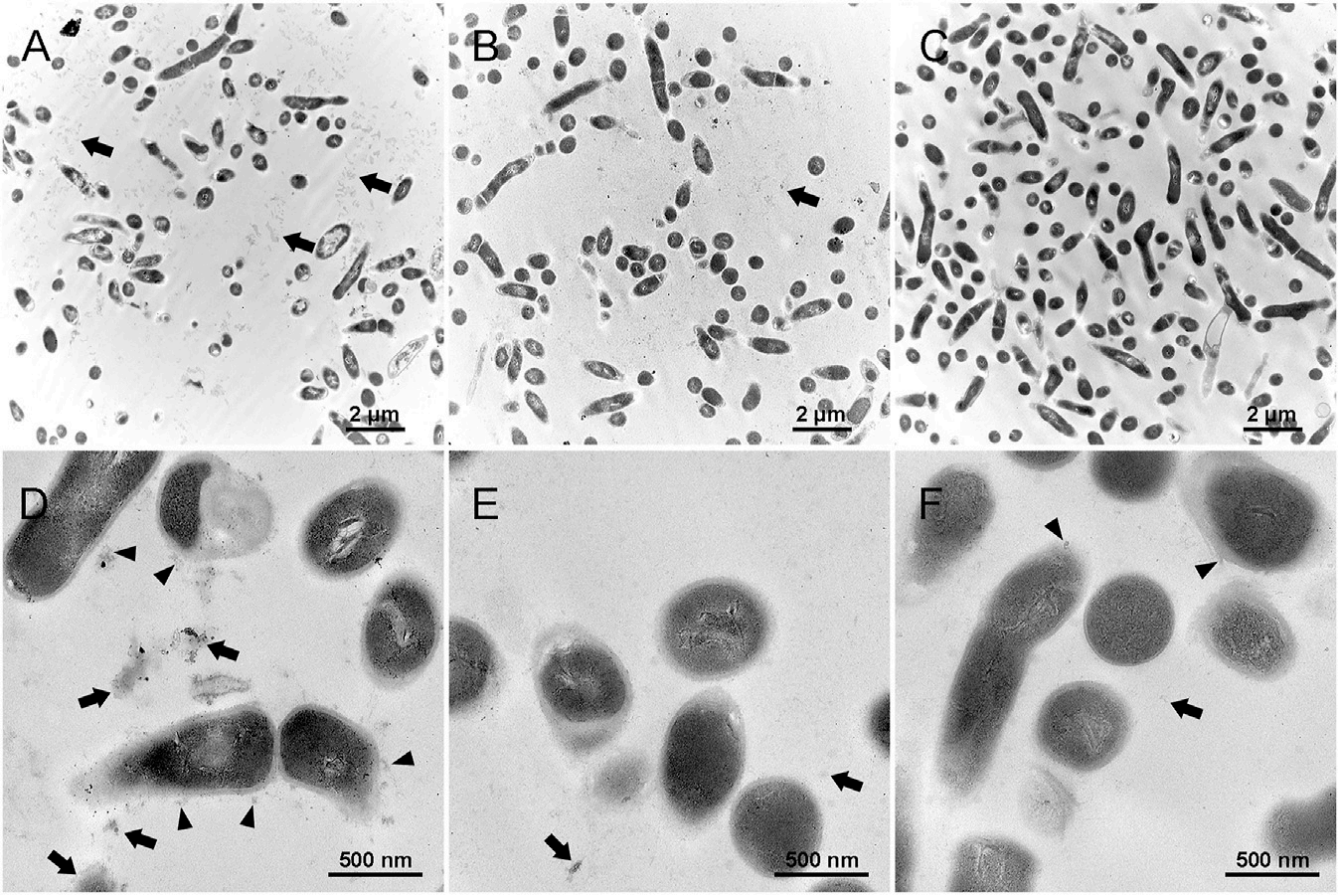
Effects of 0.01% Amy + 8 mM R on the structure of *A. viscosus* biofilm extracellular matrix under TEM. **(A,D)** The extracellular matrix was relatively massive in the biofilm of the control group. The black arrows show the extracellular matrix, and the black triangles indicate the EPS or extracellular matrix binding to the cell surface. **(B,E)** The detached substances from the biofilm that treated by 0.01% Amy + 8 mM R. The black arrows indicate the sparse detached substances, which were much less than the control group. **(C,F)** The biofilm remained on the plate after the treatment of 0.01% Amy + 8 mM R. The extracellular matrix almost vanished, few EPS that attached to cell surface could be observed (black triangles). The black arrows indicate the few extracellular matrix. No profound difference in the cell structure exists among the groups. Scale bars: 2 μm in **(A–C)**, 500 nm in **(D–F)**.

Figures 7A,D represent the biofilm in the control group, which shows that the extracellular matrix was relatively massive compared with the other groups. The black arrows show the extracellular matrix, and the black triangles indicate the EPS or extracellular matrix binding to the cell surface. Figures 7B,E represent the detached substances from the biofilm treated by 0.01% Amy + 8 mM R. The black arrows indicate the sparse detached substances, the volume of which was also much less than the control group. Figures 7C,F represent the biofilm remained on the plate after the treatment of 0.01% Amy + 8 mM R. Contrary to the first two groups, the extracellular matrix almost vanished. Few EPS that attached to cell surface were observed (black triangle). There were no profound differences in cell structure among the groups.

### Analysis of Molecular Dynamics Simulations and Molecular Docking

**FIGURE 8:**
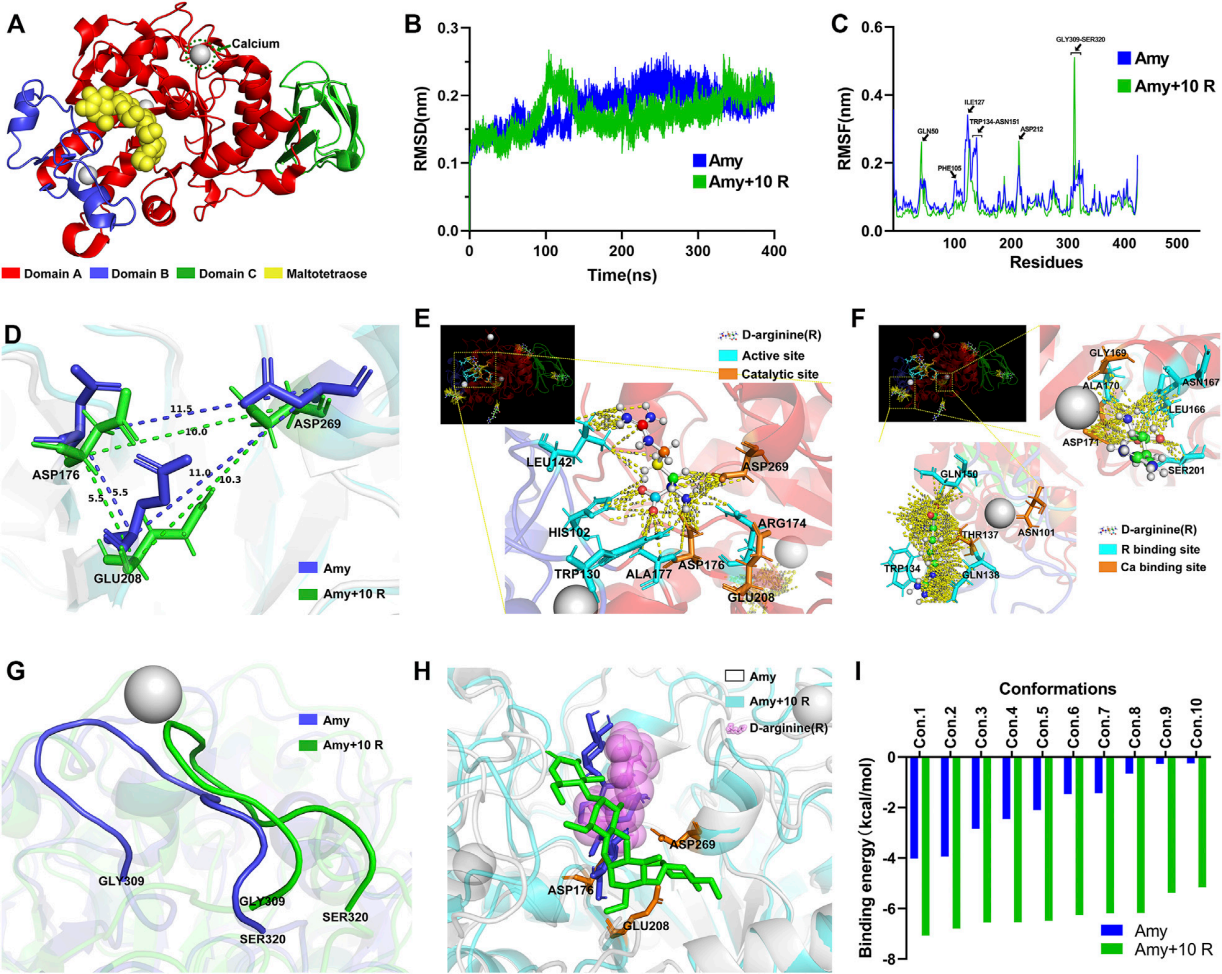
Molecular dynamics simulations and molecular docking. **(A)** Full Amy 1UA7 structure. Domain A, Domain B, Domain C, calcium ions, and maltotetraose is represented in red, blue, green, gray, and yellow, respectively. **(B)** The RMSD values of Amy and Amy + 10 R during 400 ns of simulations. Amy in blue, Amy + 10 R in green. **(C)** The RMSF values of Amy and Amy + 10 R. The residues of GLN50, ASP212, and GLY309-SER320 showed greater fluctuations in Amy + 10 R, while the fluctuations of PHE105-ASN151 residues were drastically attenuated in Amy + 10 R. Amy in blue, Amy + 10 R in green. **(D)** The distances between catalytic triad in Amy and Amy + 10 R. **(E)** The contacts between chains within 4.0 Å in the catalytic region of Amy + 10 R, represented in yellow dotted lines. The R was preset as ball and stick. Active sites were labeled in cyan, catalytic triad was labeled in orange. **(F)** The contacts between chains within 4.0 Å in the calcium-binding regions of Amy + 10 R. The calcium-binding sites as well as adjacent residues were tightly in contact with R. The R was preset as ball and stick. R binding sites were labeled in cyan, calcium-binding sites were labeled in orange. **(G)** The drastically altered conformation of flexibility in domain A, from GLY309 to SER320. Amy in blue, Amy + 10 R in green. **(H)** The superposition of the best pose of maltotetraose in complex with Amy as well as Amy + 10 R after molecular docking. Amy in white, Amy + 10 R in cyan, maltotetraose docking with Amy in blue, maltotetraose docking with Amy + 10 R in green, R was represented as translucent magenta spheres. **(I)** The docking score of each conformation in Amy and Amy + 10 R. Amy in blue, Amy + 10 R in green. Amy, *α*-amylase. R, D-arginine. RMSD, Root-Mean-Square Deviation. RMSF, Root-Mean-Square Fluctuation.

MD simulations: Figure 8A represents each domain of Amy 1UA7, the calcium ion, and the maltotetraose. Figure 8B illustrates the Root-Mean-Square Deviation (RMSD) values of Amy and Amy + 10 R. During the MD simulation, the overall RMSD values of Amy + 10 R were lower than the Amy group, indicating that 10 R might play a pivotal role in stabilizing the structure of Amy. Figure 8C depicts the differences of Root-Mean-Square Fluctuation (RMSF) between Amy and Amy + 10 R. Higher fluctuations at residue GLN50 and GLY309-SER320 in Amy + 10 R implied that these flexibilities were affected by the binding to R. While the RMSF values of residue PHE105-ASN151 were decreased, which is the calcium-binding region in Domain B. Moreover, a subtle change in the active site ASP212 could be recognized. Figure 8D illustrates the distance changes between catalytic triad (ASP176, GLU208 and ASP269). After the addition of R, the distance between ASP176 and ASP269 was shortened from 11.5 Å to 10.0 Å, while the distance between GLU208 and ASP269 was shortened from 11.0 Å to 10.3 Å. Figure 8E shows the contacts between chains within 4.0 Å in the catalytic region of Amy + 10 R. R contacted with multiple residues, including catalytic sites ASP176 and ASP269. Figure 8F shows the contacts between chains within 4.0 Å in the two calcium-binding regions of Amy + 10 R. The putative defined calcium-binding sites were tightly in contact with R. The three-dimensional adjacent residues were also closely in contact with R. Figure 8G depicts the drastically altered conformation of flexibility at domain A, from GLY309 to SER320, indicating that the flexibility of Amy might be affected by R. Supplementary Video S2 recorded the three-dimensional comparison of the structures from GLY309 to SER320 between Amy and Amy + 10 R. This conformational change was in line with the results of RMSF in Figure 8C.

Molecular docking: Supplementary Figure S2 and Supplementary Video S3 represent the position of maltotetraose in the binding pocket. Figure 8H illustrates the superposition of the best pose of maltotetraose in complex with Amy as well as Amy + 10 R after molecular docking. The maltotetraose kept closer to the catalytic sites in Amy + 10 R than Amy, which might accommodate the maltotetraose in the binding pocket with more efficacy. The docking score of each conformation was drawn in Figure 8I. The highest absolute value of Amy and Amy + 10 R was 4.02 kcal/mol and 7.07 kcal/mol, respectively. While the lowest absolute value of Amy and Amy + 10 R was 0.25 kcal/mol and 5.16 kcal/mol, respectively.

## Discussion

This study investigated the effects of R on enhancing Amy disassembling *A. viscosus* biofilm, and elucidated the theoretical molecular mechanisms. 8 mM R could prominently enable 0.01% Amy to disassemble *A. viscosus* biofilm in 30 min without cytotoxicity, and 8 mM R performed better effects on enhancing Amy hydrolyzing starch than 60 ppm Ca^2+^. Furtherly, the mechanisms for R enhancing Amy include R increased the overall stability of Amy and the activity of the catalytic triad, and R also increased the stability of calcium-binding sites.

Implants have been widely used in oral rehabilitation, artificial joints, bone fixators, and other bone-related fields (Hanawa, 2019). An untainted titanium surface is the guarantee of osseointegration (Buser et al., 2017). Nevertheless, biofilm-associated infections are the main cause of the early failure after implantation or for the PiM during the long-term period. Unlike other application fields, dental implants are threatened by bacterial infection for their whole period due to their contact with the multimicrobial oral environment. Plaque biofilm will accumulate around implant attributed to bad oral hygiene habits, food impaction, smoking, et al. Subsequently, the immune response is evoked, leading to soft tissue inflammation, bone resorption, and implant surface exposure (Fu and Wang, 2020). Hence, it is essential to eliminate biofilm to terminate the process of PiM and promote the regeneration of bone around the implants.

Eradicating bacterial biofilms has been a huge concern nowadays. However, the administration of conventional antimicrobial agents lacks efficiency since they fail to approach the massive bacteria encapsulated by the extracellular matrix (Koo et al., 2017). Thus, many studies reported effective antibiofilm agents capable of disrupting the extracellular matrix. Nanocarriers, designed positively charged, could be more likely to interact with the extracellular matrix (Fulaz et al., 2019). For instance, chitosan oligosaccharide-capped gold nanoparticles could eradicate mature *Pseudomonas aeruginosa* (*P. aeruginosa*) biofilm by electrostatic interactions (Khan et al., 2019). On the contrary, tobramycin, being positively charged, was blocked by the *P. aeruginosa* biofilm, while the neutrally charged ciprofloxacin could easily penetrate the biofilm (Tseng et al., 2013). These contradictory results indicate that nanocarriers could not penetrate biofilms with the pure electrostatic theory. Moreover, recent strategies of nanocarriers mainly focus on disrupting eDNA or proteins, not the more quantities of polysaccharides. PDT involves the use of photosensitizers, leading to the production of reactive oxygen species (ROS). ROS is considered to oxidize the cellular components, like lipids and DNA. In fact, ROS could also have the capability of attacking extracellular matrix molecules by disrupting the cell-to-cell and cell-to-matrix interactions, which causes the degradation of matrix structure (Li et al., 2013). Nonetheless, PDT also possesses the difficulty to penetrate the biofilm matrix easily and could not degrade the complex matrix rapidly (Pinto et al., 2020). Enzymes that possess the capability of degrading EPS, protein or eDNA could be employed as potential biofilm disruptive agents. Dispersin B is a well-known glycosyl hydrolase that could disrupt the major EPS of *Staphylococcus epidermidis* biofilms by specifically hydrolyzing poly-N-acetylglucosamine (Chen and Lee, 2018; Piarali et al., 2020). Deoxyribonuclease I (DNase I) is highlighted for antibiofilm purposes due to its effects on disrupting eDNA. For instance, DNase I efficiently increased vancomycin activity against the biofilm of *Enterococcus faecalis*, which could decrease the dosage of vancomycin by 8-fold (Torelli et al., 2017). In the present study, Amy showed significant effects on dispersing *A. viscosus* biofilm and hydrolyzing starch with the addition of R, implying that the Amy + R might be an optimal compound for decontaminating the biofilm-contaminated implant surface.

The PiM-associated biofilm is a cluster of definite bacterium complex. Several studies have assessed bacterial adhesion and colonization on implant surfaces through simple biofilm models or multispecies biofilm models (Schmidt et al., 2017; Vilarrasa et al., 2018; Bermejo et al., 2019). Despite the diversity of bacterial strains, *Actinomyces* is the widely used species for its initial adherence on titanium surfaces as early as 15–20 min after incubation (Guggenheim et al., 2001; Schmidlin et al., 2013). Similarly, *Actinomyces* was selected in the present study to represent the most critical species in forming the biofilm of PiM. After 48 h of incubation, the biofilm expressed compact and multi-layered (Figure 6), consistent with the study of Yamane et al. (2013). Especially, there were porous micro-pores among the biofilm, which might facilitate the exchange of nutrients and gas. A previous study demonstrated that the major components of the EPS of *A. viscosus* were: N-acetylglucosamine (62%), galactose (7%), glucose (4%), uronic acid (3%) (Rosan and Hammond, 1974). Whereas another study revealed that 39% galactose, 37% N-acetylglucosamine, 19% glucose, and 5% mannose were the main components of EPS produced by *A. viscosus* (Imai and Kuramitsu, 1983). Theoretically, N-acetylglucosamines compose poly-N-acetylglucosamine by forming *β*-1,6 glycosidic bond, galactoses compose lactose by forming α/β-D-galactose residues, and glucan, starch, as well as cellulose could be composed of glucose either by *α*-1,4 glycosidic bond, or *α*-1,6 glycosidic bond or *β*-1,4 glycosidic bond. Therefore, in order to find the feasible glycoside hydrolases for hydrolyzing the above-mentioned glucosidic linkages, Dispersin B, *α*-galactosidase, *β*-galactosidase, dextranase, cellulase, and Amy were employed. Unexpectedly, merely Amy at supersaturated concentrations showed assumed results. Amy specifically endohydrolyzes the *α*-1,4-D-glucosidic linkages in polysaccharides containing three or more *α*-1,4-linked d-glucose units. Hence, we presume that the EPS of *A. viscosus* biofilm are mainly composed of polysaccharides containing α-1,4 glycosidic bond, which is inconsistent with previous studies (Rosan and Hammond, 1974; Imai and Kuramitsu, 1983). Fleming et al. showed that 0.25% Amy reduced the biomass of *Staphylococcus aureus* and *Pseudomonas aeruginosa* polymicrobial biofilms (Fleming et al., 2017). However, the saturation concentration of Amy is merely 0.01%, higher concentrations could lead to apparent cytotoxicity (Figure 2). As a consequence, 0.01% Amy was furtherly investigated.

Most thermostable amylases require the additional Ca^2+^ for their thermostability. For instance, 5 mM Ca^2+^ enhanced the relative activity (%) of *α*-Amy from 100 to 115% (Lin et al., 1998). 1, 5 and 10 mM Ca^2+^ enhanced the relative activity of *α*-Amy from 100 to 105, 109, and 116%, respectively (El-Banna et al., 2007). The mechanism might be that the binding of Ca^2+^ ions to the *α*-helical structure of *α*-Amy increases the overall stability of *α*-Amy. Figure 4D showed that the mean relative activity of 0.01% Amy was raised from 100 to 113.0% with the addition of 60 ppm Ca^2+^, but the statistical difference was scant. Strikingly, 8 mM R dramatically increased the efficiency of Amy to 175%. Moreover, 60 ppm Ca^2+^ and 8 mM R showed a synergistic effect on enhancing the relative activity of Amy from 100 to 240%. These results indicate that R expresses a better effect than Ca^2+^ on enhancing Amy, and there is a synergistic mechanism between them. Figure 8F depicts the three-dimensional structure at the calcium-binding regions after MD simulations. There were massive hydrogen bonds between the residues of Amy and R. The formally defined calcium-binding sites also showed strong affinity with R. Additionally, the values of RMSF from PHE105 to ASN151 showed a sharp reduction with the addition of R (Figure 8C), which could be attributed to the firmly bonding interactions between R and Amy.

The results of CLSM are in line with SEM, both of them indicated that there remained few individual bacteria and sparse EPS after the treatment of 0.01% Amy + 8 mM R. Magdalena et al. reported that glycoside hydrolase (PelA_h_) reduced the *Pseudomonas. aeruginosa* cells and polysaccharide elements, remained visible bacterial cells on the membrane (Szymanska et al., 2020). Similarly, minor *A. viscosus* could be observed on the plate in this study, whereas the remaining cells were bare without the enmeshing of extracellular matrix. Bacterium could adhere to biomaterials through capsular polysaccharides, fibronectin-binding proteins, collagen-binding adhesin, lipoteichoic acid, or other surface components (Arciola et al., 2018). Likewise, TEM showed the sparse extracellular matrix on the surface of the remaining cells (Figure 7). Hence, although the EPS of *A. viscosus* biofilm were entirely eliminated by Amy + R, the initial cells adhering to the surface could not be removed easily by Amy + R for their irreversible attachment *via* active adhesion (Carniello et al., 2018). It is worth noting that 0.01% Amy seemed to increase biofilm biomass (Figure 5C), which could be interpreted by the morphology of SEM (Figure 6). Briefly, after the treatment of 0.01% Amy, there was no notable reduction of biofilm, nevertheless, several cracks and huge cavities were created to form meshwork-like structures. As a consequence, the biofilm became looser and the volume increased accordingly. With the addition of 8 mM R, the EPS were hydrolyzed more evenly. Figure 5E demonstrated that the surface to biovolume ratio of 0.01% Amy + 8 mM R was much higher than the control group as well as 0.01% Amy group, facilitating the accessibility of antimicrobial agents contacting with the cell surface. Brendan revealed that glycoside hydrolase (Sph3_h_) could potentiate the treating effects of posaconazole on *Aspergillus fumigatus via* disrupting the biofilm (Snarr et al., 2017), revealing the analogous mechanism with Figure 5E.

MD simulations predict how each atom in a protein or other molecular substance will move over time, based on experimental structural biology data (Karplus and McCammon, 2002). These simulations can capture a wide variety of important biomolecular processes, including protein folding, ligand binding, conformational changes, and revealing the positions of all the atoms at femtosecond temporal resolution (Hollingsworth and Dror, 2018). Hence, MD simulations could be exploited to determine how a biomolecular system will respond to some perturbation. A number of MD packages, such as CHARMM, AMBER, GROMACS, and LAMMPS could be used to perform biological macromolecular simulations (Lee et al., 2016). Among them, however, GROMACS might be the fastest MD package for its huge codes constantly written by the developers. In addition, the GROMACS analysis facilities for post-processing trajectories are quite extensive, and many other tools could increase a researcher’s productivity, regardless of the simulation package used. Thus, the total time to solution can be minimized by incorporating GROMACS in simulation and analysis (Vermaas et al., 2016). In this study, the molar concentration of R in parts 2.2–2.8 was 8 mM, while the molar concentration of Amy was about 6.4 × 10^–5^ mM. Therefore, the molecular amount of R was much higher than Amy. In order to optimize the MD simulation process for energy minimization, R was set decuple than Amy in part 2.9. The MD results showed that 6 R molecules interacted directly with Amy, the other four molecules were dissociated in the force field. PDB 1UA7 is a compound of Amy, containing 422 amino acids, from *Bacillus Subtilis* complexed with acarbose. The crystal structure of the Amy is divided into three distinct domains (Figure 8A), namely Domain A, Domain B, and Domain C. Domain A (PRO4-ILE100, THR152-LEU352) comprises the typical (α/β)8-Barrels, which is the catalytic domain. Domain B (ASN101-ASN151) is a short loop ring structure extending from Domain A, characterized to hold tightly to a calcium ion, thus being pivotal to the stability of protein. Domain C (SER353-ASP425) comprises a typical antiparallel *β*-sheet structure. McCorvy et al. (2018) showed that the modified indole-aripiprazole hybrid compounds uncovered the ligand egress of G protein-coupled receptors, indicating the positive response of the receptor to ligand. Dexamethasone privileged only a few poses for the glucocorticoid receptor, providing high rigidity to receptor-ligand complex, thus suitable for recognizing substrates (Alves et al., 2020). Likewise, the RMSD values were reduced and the absolute values of docking scores were significantly improved with the addition of 10 R, implying the potential functional changes of Amy affected by R. Metal-organic frameworks (MOFs) with the shortest inter active site distance (15.6 Å) showed the highest record apparent quantum efficiency in good accordance to biological systems, indicating that a smaller distance leads to higher activity (Gong et al., 2020). In the same vein, greater distances between the catalytic triad in mutant *Solanum tuberosum* resulted in the prevention of hydrogen bonding which is critical for catalytic activity (Hussain and Chong, 2017). In the present study, similarly, the distances between the catalytic triad were shortened by the binding of the catalytic region to R (Figures 8D,E), which consequently potentiated the catalytic activity of Amy. Flexibility is a key feature of proteins to maintain local changes in conformation (Hollingsworth and Dror, 2018). Figure 8G illustrates the most notable conformational change of Amy with the addition of R, corresponding with the highest peak in RMSF, implying that this random coil from GLY309 to SER320 might reflect the overall stability of Amy.

Comprehensively, based on the results of biofilm biomass, EPS hydrolysis, biofilm morphology, extracellular matrix structure, MD simulations, and molecular docking, 8 mM R could optimally enhance the disassembly effects of 0.01% Amy on *A. viscosus* biofilm. Still, there exist some limitations. For instance, the effects of Amy + R on the biofilms cultured on the titanium surface should be further investigated. In addition, the feasibility of Amy + R on treating multispecies biofilms should be verified in the next phase of development.

## Conclusion

In summary, this study demonstrates that D-arginine may enhance the disassembly effects of alpha-amylase on *Actinomyces viscosus* biofilm through potentiating the catalytic triad as well as stabilizing the calcium-binding regions, thus providing a novel strategy for the decontamination of biofilm contaminated implant surface.

## Data Availability

The raw data supporting the conclusion of this article will be made available by the authors, without undue reservation.
