# Supplementary figures and images for: D-arginine Enhances the Effect of Alpha-Amylase on Disassembling Actinomyces viscosus Biofilm
Source: Front Bioeng Biotechnol. 2022 Mar 3;10:864012. doi: 10.3389/fbioe.2022.864012 (PMC8927782; doi:10.3389/fbioe.2022.864012)

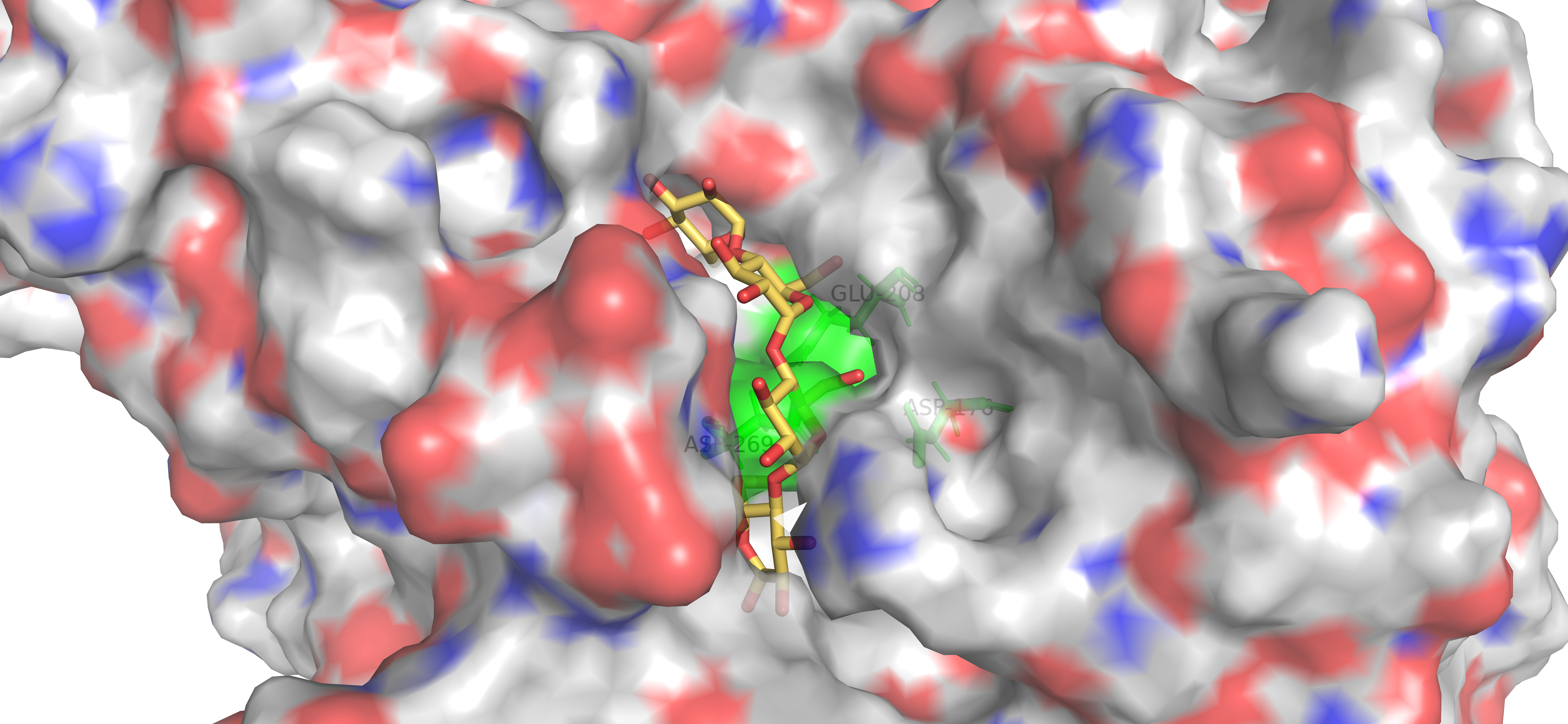

Supplement: Supplementary file 2 [file Image2.TIF]

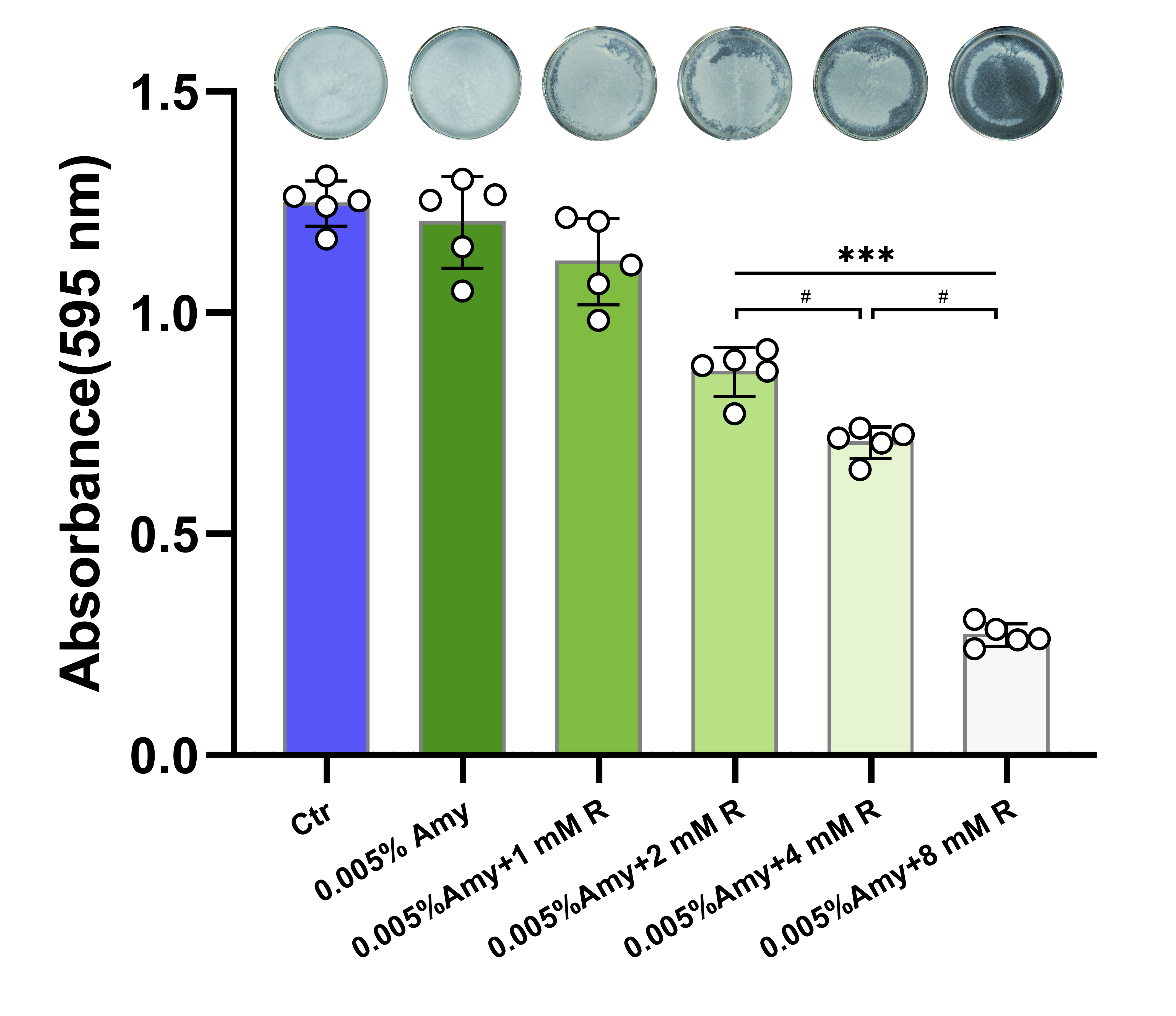

Supplement: Supplementary file 3 [file Image1.TIF]
